# Milk Products from Minor Dairy Species: A Review

**DOI:** 10.3390/ani10081260

**Published:** 2020-07-24

**Authors:** Michele Faccia, Angela Gabriella D’Alessandro, Andrea Summer, Yonas Hailu

**Affiliations:** 1 Department of Soil, Plant and Food Sciences (DiSSPA), University of Bari, Via Amendola 165/A, 70126 Bari, Italy; 2 Department of Agricultural and Environmental Sciences (DiSAAT) Via Amendola 165/A, 70126 Bari, Italy; angelagabriella.dalessandro@uniba.it; 3 Department of Veterinary Science (DSMV), University of Parma, Via del Taglio 10, 43126 Parma, Italy; andrea.summer@unipr.it; 4 School of Animal and Range Sciences, Haramaya University, P.O. Box 138, Dire Dawa 3000, Ethiopia; yonashailu421@gmail.com

**Keywords:** minor dairy species, milk processing, dairy products, equine, camel, yaks

## Abstract

**Simple Summary:**

Dairy products represent an important food source for worldwide people. The milk used for their manufacturing is mostly supplied from the four major ruminant species (cow, goat, sheep, and buffalo), on which the research has been focused for long time. In recent years, the social transformation underway in poor and developing countries, climatic changes, and increased attention to animal welfare are shining a light on some minor animal species that have a “local dairy importance” such as equines, camels, and yaks. Even though not specifically reared for milk production, they are sometimes regularly milked for the manufacturing of a series of dairy products such as fermented milk, cheese, butter, and other fat- and non-fat-based specialties. The investigations on their manufacturing processes, and on their chemical-nutritional and microbiological properties, are now rapidly increasing, and a robust set of scientific data is beginning to form. These information will help to improve the quality of these products and to spread the knowledge about them all over the world. In the near future, they could represent a source of innovative and functional foods for a wider number of people, supplying an income to farmers and enhancing food biodiversity.

**Abstract:**

Milk processing is one of the most ancient food technologies, dating back around 6000 BC. The majority of dairy products are manufactured from cows, buffaloes, goats, and sheep; their production technologies are mostly standardized and have been widely investigated. Milk and dairy products from minor species are less important under the economic point of view, but they play a fundamental social role in many marginal and poor areas. Due to scarce interest of the dairy industry, their technological characteristics and related issues have been investigated less. Recently, the increasing interest toward ethnic foods and food biodiversity is helping these minor products to emerge from the “darkness” in which they have remained for long time. Some of them are increasingly seen as useful for the valorization of marginal areas, while others are recognized as innovative or healthy foods. The present review aims to resume the most recent knowledge about these less-known dairy products. The first part summarizes the main technological properties of equine, camel, and yak milk with a view to processing. The second is a survey on the related dairy products, both the traditional ones that have been manufactured for a long time and those that have been newly developed by food researchers.

## 1. Introduction

Today, cattle produce 82% of total world milk, the remaining share is mostly obtained from buffaloes, goats, and sheep. Less than 1% derives from other animals, such as camels, horses/donkeys, and yaks [1], which can be defined as “minor dairy species.” For a long time, dairy research has focused on bovine milk, and the other three major ruminant species were approached by researchers with the progress of the dairy industry, the increase in demand of non-cow dairy products, and the subsequent marketing organization. The minor species have low economic significance, and consequently, the research on them has only recently started: scientific information about mare and camel milk date roughly to less than 30 years, whereas for other species, such as donkey and yak, the investigation significantly increased in the last 20 years. The increasing interest on minor dairy species was highly related to the social role that they play in the geographical areas in which they are reared. A significant number of publications on minor dairy products are now available, and it is clearly emerging that they represent not only a priceless cultural heritage but also a source of innovative and, in some cases, healthy foods. Most of the relevant studies deal with the management of lactation and the chemical-nutritional properties of milk, but recently, they have also focused on dairy processing. 

At the best of our knowledge, a comprehensive review on dairy products from minor species is not yet available in the scientific literature. The aim of the present work was to close this gap by performing a state-of-the-art of the research on milk products from equines, camels, and yaks, with an insight also on other species defined as “marginal,” since they are only occasionally milked and involved in dairy processing. The review is divided into two parts: the first one describes the main technological properties of milk, and the second is a survey of the related dairy products and their chemical and nutritional characteristics.

## 2. Technological Properties of Milk

### 2.1. General Aspects

For dairy processing, the aptitude of milk to form a gel by acidification or renneting is pivotal. Acidification is mostly used for manufacturing fermented liquid products, and any type of milk undergoes at least a slight increase of viscosity when the isoelectric point of caseins is reached. For fermented milks, the main technological issues are the acidification time and the obtainment of the required level of viscosity, whereas making cheese involves a much more complex approach. Most of the cheeses known worldwide are made by enzymatic coagulation, which requires two fundamental properties—coagulability and cheese yielding capacity. Coagulability is the aptitude to form a firm coagulum upon the addition of proteolityc enzymes, with suitable retention of milk solids. It can be assessed by different techniques, but the most common is based on the use of a lactodinamograph, an instrument that performs a mechanical measurement. The formagraph is the most popular lactodinamograph (Foss Electric A/S, Hillerød, Denmark). The three “classic” parameters measured by this instrument are the rennet coagulation time (RCT), curd firming time (k20), and curd firmness (a30) throughout a 30 min period [2]. These parameters have been widely studied in cow milk, and iit s well known that milk with short RCT and curd firming time and good curd firmness is expected to give more cheese with desirable composition than milk with unfavorable properties [3,4,5]. The cheese yielding capacity, expressed as kg of cheese obtained from 100 kg milk, is highly connected to the “compositional richness” of milk—in particular, in dry matter and casein. Yield can be predicted by applying specific formulae that have been developed by several researchers: originally, they were only based on total protein and fat content of milk, but other parameters were included with time. In particular, it has been demonstrated that the casein polymorphism connected to genotype, season, stage of lactation, and parity of the cows also make an important contribution [6,7,8,9,10]. The matter is under continuous investigation, and some physical aspects, such as the size of casein micelles and of milk fat globules, have been considered. The micelle size has a dominant effect on coagulation time and curd firmness; the strongest rennet gels were obtained when small casein micelles (153–159 nm) were combined with large fat globules (3.88–5.78 μm) [11]. The fat globules are greatly important in cheese because they prevent the casein micelles from completely coalescing into thicker strands; a high fat content and its even distribution into the curd provides a smooth texture [12]. In minor dairy species, the physical status of fat globules and casein micelles are still under study, even though sufficient information are already available. This aspect must be carefully considered in dairy processing, since the size of these particles is different in minor milks with respect to the “major” ruminants. Table 1 summarizes the mean values of the main compositional aspects of minor milks that can play a primary role in dairy processing, such as the dry matter content, the casein profile, the physical-chemical characteristics of the casein micelle and fat globule, the casein/whey proteins ratio (Cn/Wp), and the calcium equilibria.

Somatic cell count (SCC) counts (TBC) are the other two important quality parameters of milk destined for dairy processing. The study on these subjects on minor milks is far from being considered concluded, but many studies are already available. A comprehensive reviewing of this information is beyond of the scope of the present work, but some basic indications will be given. It is known that EU legislation contemplates a maximum level for SCC only for cow milk (400,000 cells mL^−1^), whereas the TBC limits are different for cows (100,000 cfu mL^−1^) and other ruminants (500,000 cfu mL^−1^ or 1,500,000 cfu mL^−1^, depending on the presence of heat treatment before processing). These latter values are also valid for any other type of milk.

### 2.2. Equine Milk

Today, the world horse population is over 60 million, mostly reared in America and Asia, whereas in Europe, they are mostly present in the eastern regions. The total donkey population is estimated to be about 56 million, mainly concentrated in Africa and Asia. In the EU, there are around 288,000 donkeys, most of which live in Spain, France, Romania, and Italy [1]. In poor and developing countries, equids are used mainly as transport animals, while in developed countries, they are reared for several purposes, including the production of milk, particularly in Europe. The chemical and nutritional characteristics of horse and donkey milk are similar, and both differ considerably from that of the principal dairying species. In comparison with cow milk, they contain less total solids, fat, and protein and more lactose, showing a composition close to that of human milk [13,14,15,16,17,18,19]. In general, the poor dry matter content makes gelification difficult, but other concerns derive from the composition of the protein fraction. In particular, Cn/Wp is less than one third with respect to cow milk; the casein micelle is larger and contains a different pattern of single fractions, with β-casein being the most abundant and k-casein present in trace amounts (Table 1). However, recent research is supplying evidence of a wide genetic variability in synthetizing the αs1 fraction, suggesting that the casein pattern can present wide variations from individual to individual [20,21,22]. The scarce presence of k-casein is responsible of higher average size of the micelle and different mechanisms for micelle stability with respect to cow milk [23,24,25]; these characteristics, together with the low total casein content, are probably involved in the poor coagulation properties. The zeta potential (one of the most used indices for measuring the stability of colloidal dispersions) of micelles has been reported for donkey milk and is lower than cow milk (−20.5 mV versus −15.4 mV); this difference, together with the low hydration level, was hypothesized to be responsible for the poor thermal stability of equine milk [26]. Despite this low colloidal stability, poor gelification is observed both for acidic and enzyme coagulation, despite the fact that the availability of soluble calcium is higher in equine milk than in bovine milk. Uniacque-Low reported that horse casein micelles were not coagulable by calf chymosin at pH 6.6 and that some micellar flocculation occurred in the acidification of milk but no gelation [27]. Pochet et al. [28] investigated the effect of different enzymes on horse milk coagulation under the influence of several technological parameters, including concentration by ultrafiltration, heat treatment, adjunct of calcium chloride, renneting pH, and temperature of coagulation. Some “flakes” were formed when the enzymes were added, but the milk never actually coagulated to form gel, which prevented any significant signal to be measured by Formagraph. The visually observed “coagulation” time decreased with acidification and increased with temperature and amount of coagulant; bovine rennet with high pepsin content gave better results than fungal protease. The addition of calcium chloride above 0.05 g/L and the concentration of proteins by ultrafiltration had no effects, whereas heating the milk worsened coagulation. In another study, equine milk did not show rennet coagulation even after 2 h in the presence of Ca^++^, but when cow k-casein was added, a weak coagulum occurred in the long term; unfortunately, the authors did not supplied the exact time needed for coagulation [29]. A vegetable extract from the fruit of *Withania coagulans* coagulated milk, but the details of the physical and chemical properties of the curd obtained were not reported [30]. The effectiveness of animal rennet in hydrolyzing the casein fractions of horse milk has been investigated by several researchers. Calf chymosin only produced peptides from β-casein, whereas κ-casein was not degraded over 24 h at 30 °C and pH 6.5 [31]. A study conducted on pureβ-casein demonstrated that the bond at Leu190-Tyr191 was readily hydrolyzed [32]. As to donkey milk, several authors reported the same difficulties in gelification, but a weak coagulum was obtained by adding different enzymes (camel chymosin, calf and microbial rennet, and cyprosin). It was reported that the reason of poor firmness is attributable to the secondary phase of coagulation (aggregation of the hydrolyzed micelles) rather than to the enzymatic phase [33,34,35,36]. By electrophoresis, it was found that the microbial coagulant from *Mucor mihei* released a series of fragments that seemed to correspond to γ-casein-like and β-I- and β-II-caseins-like fragments from horse β-casein; two overlapping bands with high electrophoretic mobility were hypothesized to derive from αs1-casein, whereas two fragments having intermediate electrophoretic mobility remained unknown [35]. Also for donkey milk, the lactodinamograph failed to measure the coagulation parameters due to irregular formation of the clots, and it was suggested that a dedicated procedure should be developed by modifying the probes of the instrument [34]. 

Few studies are available in the literature regarding somatic cell content and total bacterial counts in equine milk (Table 2). In general, the studies agree that the values are rather low, and for SCC, they are lower than cow milk. Pecka et al. [37] reported that the increase in somatic cell count in horse, as in cow milk, is connected with a decreased level of the components synthesized by galactopoietic cells of the udder and with an increase in the level of secretion of whey proteins. As in cows, the inflammatory state of the mammary gland is the main reason for the increase in SCC in milk [37,38], but other authors have suggested that, while in cows, leucocytes contribute to the defense against invading pathogens, the immune system in horses may act differently in connection with the high concentration of lysozyme, lactoferrin, and immunoglobulin. Further studies are needed to establish a pattern of somatic cells in horse milk and to determine whether pathological and non-pathological factors influence it [39]. As considered by Kaić et al. [40], mares have a generally good health status of the mammary gland and good microbial quality of milk due to the low volume of udder, high resistance to pathogens, and high concentrations of antimicrobial compounds; in addition, having not been selected for milk yield, they are less sensitive to inflammation and infections. Costa et al. [41] report that, in Brazil, mastitis affects 5–10% of the herd of breeding mares, usually in the period of involution of the mammary gland, and it may cause impairment in weight gain of suckling foals, especially after a clinical mastitis; nevertheless, they concluded that milk has better hygiene properties (SCC and microbial count) compared to bovine milk, and this can be used as a marketing strategy when selling milk for human consumption. The SCC values reported are highly variable: the highest one was reported in Croatian Coldblood Horse (10–47 × 1000 mL^−1^) and in Thoroughbred and Polish Half Bred (20–37 × 1000/mL), whereas the lowest value of 3.02 × 1000 mL^−1^ was registered in Slovenian Draft Horse [39,42,43,44,45,46]. Also, donkey milk is characterized by a low somatic cell count, with an average value around 35 × 1000 mL^−1^ [47,48,49]. As to the total bacterial count, the literature data indicate values ranging from 7 cfu mL^−1^ to 90,000 cfu mL^−1^, both for horse and donkey milk (Table 2), and it is generally considered that they do not frequently harbor foodborne pathogens due to antimicrobial properties connected to substances such as lactoferrrin, lysozyme, immunoglobulins, and lactoperoxidase [34,50,51,52,53,54]. However, hygienic issues have sometimes been reported [53]. For example, Zhang et al. [51] reported that even though *Salmonella choleraesuis* and *Shigella dysenteriae* showed a certain sensitivity to natural antimicrobial compounds in donkey milk, the growth of most micoorganisms was not affected by them. The authors concluded that hygienic practices and regulations, such as on-site pasteurization and implementation of Hazard Analysis and Critical Control Point HACCP protocol, should also be introduced to facilitate to production of donkey milk of high quality and safety. In a survey of 90 samples of donkey milk, two samples contained two main food-borne pathogens of growing importance for human health: one sample harbored *Escherichia coli* O157 producing the Shiga-like-toxin (SLT-I and SLT-II), and the other one contained *Campylobacter coli*. The study demonstrated that raw donkey milk might represent a realistic health risk for consumers, and, as for raw cow milk, specific microbiological parameters and criteria need to be established for authorized dairy donkey farms [50]. The effectiveness of in-vat pasteurization at +65 °C for 30 min and storage at refrigeration (+3 °C ± 2 °C) and freezing temperatures (−20 °C ± 5 °C) on some hygienic characteristics of donkey milk was investigated. The treatment ensured compliance with EC Regulation No 1441/2007, as *Enterobacteriaceae* were never found in the milk, and TBC at 30° never went beyond 1 log cfu ml (initial load was 4.84 log cfu mL^−1^) [55]. A study on 101 half-udder milk samples showed very low values of TBC (<250 cfu/mL) and a minor presence of pathogens: *Staphilococcus aureus* was isolated from only five milk samples (three animals), *Streptococcus equi* from two samples, and *Streptococcus equisimilis* from a single sample. None of the *S. aureus* isolates harbored genes coding for any enterotoxin, toxic-shock syndrome toxin, or antibiotic resistance. The results of the study demonstrated the low incidence of intramammary infections in donkey milk and the absence of foodborne pathogens, suggesting that donkey milk could be a safe food, if the mammary gland is healthy and the animals are milked in proper hygienic conditions [47]. According to Colavita et al. [56], hazard exposure associated to the consumption of raw horse and donkey milk has to be considered lower than for cow milk, especially for microorganisms like enterotoxigenic *E.coli* and thermo-tolerant *Campylobacter*. Thermal treatment before consumption is always recommended, in particular in those regions of the world where horses, donkeys, and mules are crucial components of micro-economies and the prevalence of *Brucella* spp. and *Rhodococcus equi* is higher.

### 2.3. Camel Milk

Three types of camel are reared in the world—the Bactrian camel (*Camelus bactrianus)*, the Dromedary (*Camelus dromedarius*), and their hybrids. The total population in 2018 was estimated to be around 30 million; more than 80% live in Africa, with 60% in the Horn of Africa (in particular, Somalia) [1]. The Bactrian species is domesticated in China, Asia Minor, and southern Russia, including Mongolia and Kazakhstan, whereas the Dromedary species is concentrated in the Middle East, North and East Africa, southwest Asia, and Australia. [1,65]. Camel milk represents an important food source for many people living in these regions, and its chemical composition has been widely investigated.

Literature data show a wide range of variation of the gross composition, but the average values of the most important chemical parameters are not very different from that of cow milk (Table 1). Nevertheless, the Cn/Wp ratio and the casein profile are strongly different; the latter is characterized by the presence of β-casein as the most abundant fraction, followed by αs1 and αs2, whereas κ casein is only a minor compound (less than 5% of total fractions). In addition, the average size of the fat globules and casein micelles is the opposite than cow, the former being smaller and the latter greater [66]. As a consequence, the technological properties of camel milk are very far from that of cow milk, as demonstrated by its poor ability to creaming and scarce rennet coagulation aptitude. In fact, rennet addition does not produce a firm curd, and most of the fat is lost into the whey; in some cases, only the formation of flakes has been observed [67]. The unfavorable size of the micelles and globules, the different colloidal properties, and the poor presence of k-casein are the main causes of these poor technological properties. As previously reported for equine milk, difficult gelification is probably in connection with difficulties in the secondary phase of coagulation. In fact, the primary phases are rapidly performed both by bovine and camel rennet, which hydrolyze k-casein at the Phe97-Ile98 site [68]. Literature data about the response of camel milk to coagulant enzymes are shown in Table 2. Wangoh et al. [69] separated camel rennet into two fraction by IDF method 110A, and the second fraction was more effective than the calf counterpart in coagulating camel milk. Since the second fraction is known to contain pepsin, it was hypothesized that this enzyme was more suitable than chymosin, and that camel rennet had higher pepsin content. Unfortunately, pepsin concentration was not determined, and the hypothesis done was supported by the evidence that pure porcine pepsin was five times more effective in coagulation than bovine chymosin. Renneting of 1 L of skimmed camel milk at 35 °C with 0.1 mL of a liquid rennet solution (520 mg/L of chymosin) caused the formation of small casein flakes dispersed in mellow phase. Turbidimetry failed to measure the coagulation kinetic, but it indicated a very short time (less than 1 min) for performing the primary phase (hydrolytic step) [70]. Other enzymes such as calf rennet and microbial rennet from *Endothia parasitica* were much less effective than pepsin: this finding was associated to the particular affinity of pepsin for camel milk, the incidence of environmental factors (pH, temperature, and ionic strength), or to the presence of specific protease inhibitors for the other enzymes [67]. Other authors reported that milk was coagulated in 6–8 min at 36 °C by rennet containing camel chymosin, and no significant effect was exerted by the addition of ionic calcium and by variation of pH in the range of 5.75–6.25 [71]. Hailu et al. [72] reported that the gelation time induced by camel chymosin was dose-dependent at 30 °C (a minimum time of 526 s was observed in the presence of 85 IMCU L^−1^ concentration) but was independent at 40 °C. Lowering the pH also reduced the gelation time, whereas the effect of CaCl_2_ addition was pH-dependent, since a significant improvement was only found at pH 6.3. In contrast, a positive effect of CaCl_2_ on rennet coagulation at pH 5.5 was observed by Qadeer et al. [73], who reported that the addition at 0.06% level resulted in the higher yield improved coagulation time and texture of the coagulum. In short, rennet coagulation of camel milk is difficult but can be somehow obtained by using strong dose of coagulant and suitable operating conditions that need to be better addressed, being conflicting in the literature. It should depend on the extreme variability of milk composition, with particular emphasis on total solids and casein content.

Several values of somatic cells and total bacterial counts of camel milk reported in the literature are shown in Table 2. SCC and TBC of bulk tank milk of dromedary camels kept under intensive management were investigated, and it was found that raw milk of excellent microbiological quality can be produced, but SCC levels could exceed the threshold of acceptance set for bovine milk. Relevant variations in both SCC and TVC throughout the year were observed and were associated with seasonal changes in production levels and stage of lactation [57]. In a study of 33 lactating dromedary she-camels using the California mastitis test, an overall 24.24% prevalence of sub-clinical mastitis was found, but no significant association with age, lactation stage, parity, and quarter was observed. A significant increase of electrical conductivity and pH was detected in milk from infected animals, together with lower values of protein, lactose, and fat contents [58]. A comparative study performed on 38 dromedary camels and 48 cows from Tunis reported that the mean SCC in milk were similar. In milk with low SCC, macrophages were the most represented cell (~66% of total) in camel milk, whereas lymphocytes were the most represented in cow milk (~78%); at a high SCC level, in camel milk, the relative proportion of macrophages and polymorphonuclear neutrophils increased significantly, while cow milk had an increased proportion of polymorphonuclear neutrophils and decreased level of lymphocytes [59]. Famy and Mohamed [60] reported that regular milk samples (taken at mid lactation) had significantly higher mean SCC values than milk from early and late lactation. In this milk, a strong significant positive correlation with total nitrogen, whey protein and casein, and a negative one with NPN, was found. A survey on the sanitary conditions of lactating dromedary she-camels in Egypt reported low TBC values in milk (7100 cfu mL^−1^ average value) and concluded that the milk hygienic quality was influenced by post-milking environmental contaminants rather than by animal infections [61]. The growth behavior of three strains of *Staphylococcus aureus*, *Listeria monocytogenes*, *E. coli* O157:H7 and *Salmonella* spp. inoculated in raw and pasteurized camel milk was investigated by using inoculated pasteurized bovine milk as control. All investigated pathogens grew less well in raw and pasteurized camel milk than in the control, and the inhibitory effect was more bacteriostatic than bactericidal. This effect was exerted for about 8 h both at 25 °C and 37 °C, leading to reductions in the counts of *S. aureus* and *Salmonella* spp. by approximately 1 log10 with respect to bovine milk [62]. Of course, the microbiological quality is strictly connected with the environment, including hygienic conditions of the farms and environmental temperature. In a survey conducted on 33 samples of milk from Saudi Arabia, the mean TBC count was 100 × 1000 cfu mL^−1^; *Staphylococcus aureus* was found in 70% and *Salmonella* in 24% of the samples. Results indicate the potential health risk of consuming raw camel milk under the local production conditions. *Listeria* spp. occurred in 1% of raw camel milk samples in Egypt and in 2.1% of raw camel milk samples in Iran; *Staphylococcus* spp. in samples of raw camel milk in Kenya gave mean counts of 5.2 log cfu mL^−1^ [63,74].

### 2.4. Yak and Other Milks

Yak (*Bos grunniens*) milk is predominantly produced in the Qinghai-Tibet region of China, where the animal is well adapted to the low-temperature and hypo-oxygen extreme conditions. The China yak population is approximately 13 million, corresponding to more than 90% of total in the world [75]. There is no official information about the yearly world production volume of yak milk, and estimations are very variable, ranging from 0.7 to 40 million tons [76,77]. The main differences with respect to cow milk consist of higher dry matter, fat, and protein content, whereas the casein profile is not very different, except for slightly higher and slightly lower concentrations of β and κ fractions (Table 1). However, the casein micelle is more hydrated and richer in colloidal calcium phosphate, and the average size is only slightly larger; also, the average diameter of fat globules is not very different [78,79,80]. As a consequence, rennet coagulation is easily obtained, differently from the other minor milks. The ability of bovine chymosin to coagulate yak milk was investigated by Zhang et al. [80] in comparison with cow milk—the enzyme was able to hydrolyze k-casein, but the formation of the gel was relatively slow. Nevertheless, the higher level of colloidal calcium phosphate provided a relatively firmer gel structure, in which the casein micelles were linked together in multiple directions in a closely packed form; in contrast, the gel network formed by cow casein micelles was only linked along a single direction, and the strands were partially fractured. The larger size and higher hydration of the yak micelle has been associated to decreased curd syneresis and higher moisture retention [79,80,81]; however, it was reported that the coagulation properties of yak milk were more favorable with respect to Holstein milk due to the higher casein and calcium concentration [82]. Zhang et al. [83] reported that yak milk was suitably coagulated for producing hard cheese of good sensory quality by adding 3% lactic acid bacteria starter, 30 μL/100 mL rennet, and 0.3% CaCl_2_.

Information about the relationship between mastitis and SCC, and about total bacterial count of yak milk are rare (Table 2). Increases in the concentrations of proteins of blood serum origin, including lactoferrin, were identified in milk whey from mastitic animals, while concentrations of the major whey protein were downregulated; unfortunately, somatic cells were not investigated [84]. The average TBC value of fresh yak milk was found to be around 380,000, but the sanitary quality was poor, as evidenced by relatively high numbers of coliforms (*E. coli* and *S. Aureus*) [85]. A few reports exists on the main spoilage and pathogenic microorganisms. Coliforms in raw yak milk produced in China were reported to range between 3.0 and 3.5 logcfu mL^−1^, with *E. coli* being detected in 29 samples at 2 log value. In the same samples, *Staphylococcus aureus* was identified at a maximum population of 3.5 log cfu mL^1^ [74]. High occurrence of this latter microorganism (in 50 out of 72 milk samples) was also found by Tsegmed et al. [64]. Hygienic risk associated to foodborne pathogens in raw yak milk and cheese was reported: 31 shiga toxin-producing *E. coli* (STEC) and six enteropathogenic *E. coli* (EPEC) of different serogroups and considerable genomic diversity were isolated in Arunachal Pradesh and Sikkim. Considering that the majority of the strains were resistant to a wide range of antibiotics, serious public health concern for people remaining in close proximity of yaks or consuming raw or undercooked milk were signaled [86].

Milk from other mammals is occasionally processed into dairy products, such as reindeer, moose, llama and alpaca. Hints on the technological properties of reindeer and alpaca milk have been found—reindeer milk is very dense, being particularly rich in protein and fat matter, but its fine composition is not yet very well described the fat globule size is similar to that of cow milk [87]. Calf chymosin readily coagulated it, but no information is available on the kinetic and hydrolysis sites, since the sequence of reindeer k-casein is not known, and there is no information on reindeer chymosin [88]. Alpaca milk destined to a cheesemaking trial was obtained by manual milking at the beginning of lactation It had 1.0469 ± 0.0025 density, 16.92 ± 1.74°D titratable acidity, 4.12 ± 0.08% fat, 6.34 ± 0.41% protein, and 16.84 ± 0.70% total solids [89]. Even though the suitability to enzyme coagulation needs to be deepened, a potential good aptitude to cheesemaking can be drawn not only from the high protein content but also from the proportion of casein to total protein, which was reported to be very close to that of cow (72–73%) during the first two months of lactation [90]. No technological information is available for moose and llama.

## 3. Dairy Products

### 3.1. Equine Fresh and Fermented Milk

The consumption of horse milk is widespread in the world with 30 million people that drink it more or less regularly. Dairy herds are mainly located in the former USSR and in Mongolia. Kazakh breeds are probably the most significant, having been selected over many years for milk production, while, in Western Europe, the main dairy breed is Haflinger [91]. Differently, no selection has been carried out for donkey milk production, and there are no specialized dairy breeds. Milk from both the species has historically held a special place. In particular, horse milk is a traditional food for the nomadic pastoral populations of Asia, while donkey milk was widely known in ancient popular tradition as a substitute for breast milk for infants. It is only recently that the donkey and horse were considered as minor dairy species, and studies are addressed to define the most appropriate management in an intensive production system. The functional peculiarities of horse milk, linked to protein content, casein concentration, distribution of di- and triglycerides, and proportion of polyunsaturated fatty acids have led to it being considered more suitable for human nourishment than cow milk [17]. It is also considered a possible substitute of cow milk for children affected by allergy to cow milk proteins due to its similarity to human milk, palatability, and low allergenic properties [92,93,94,95,96,97,98,99,100]. In addition, several health benefits have been attributed to *Equidae* milk consumption—horse milk has been used for the treatment of some pathologies, such as hepatitis, chronic ulcers, and tuberculosis [101,102], while donkey milk is efficient in releasing anti-inflammatory interleukins in upregulating the immune response in aged hosts, in preventing atherosclerosis, and as an anti-proliferative and anti-tumor agent [103,104,105]. All these properties have led equine milk to be considered a functional/nutraceutical food suitable for sensitive consumers and have stimulated growing scientific and entrepreneurial interest in milk for human nutrition [98,106,107,108,109], as well as in manufacturing dairy products.

The most famous dairy product obtained from horse milk is *koumiss*, also called *kumis*, *airag*, or *chigee*. It is a traditional lactic-alcoholic beverage that has been produced since around 2000 BC and is best known and widely consumed in the central Asian regions, in some regions of Russia and China, and in Mongolia. It is conventionally obtained from fresh raw milk inoculated with a bulk starter (mainly lactic acid bacteria and yeast) at 20–30% dose and kept in a suitable animal hide bag for fermentation at, usually, 20–30 °C [110,111]. The major limitation for its production is the scarce availability and high cost of mare milk, so methods have been defined to substitute it with cow milk after modification by rendering its composition similar to mare milk (dilution, removal of fat, lactose addition, filtration by membranes) [112]. The manufacturing process of *koumiss* is now more standardized, with use of wooden vats, plastic barrels, or stainless steel tanks in place of the leather containers, selected starters, and control of the inoculation temperature. Different microorganisms are involved in koumiss fermentation, the most important of which are lactobacilli (*Lactobacillus. delbrueckii, bulgaricus,* and *acidophilus*) and yeast (*Kluyveromyces, Saccharomyces, Candida*), but a wide biodiversity has been found in traditional koumiss [113,114,115,116,117]. Fermentation results in 0.6–3% ethyl alcohol, 0.5–1.5% lactic acid, and pH between 3.3 and 5.0; the sugar content is in the range 2–4%, and fat is about 2% [91,113,116]. With reference to the amount of lactic acid and ethanol produced, the product is generally recognized as light (pH 5.0–4.5), moderate (pH 4.5–3.9; 50% average lactose conversion to lactic acid), or strong (pH: 3.6–3.3; 80–90% lactose conversion) [114]. Besides being converted to lactic acid and ethanol, lactose is also the source of carbon dioxide, causing slight sparkling taste, and other byproducts such as volatile acids and alcohols, which contribute to the characteristic flavor. During fermentation, caseins undergo hydrolysis (about 10% of the total); the concentration of some amino acids (proline, serine, alanine, valine, leucine, and histidine) and some water-soluble vitamins increases. As to the sensory characteristics, *koumiss* has milky-bluish-white color with a pinkish tinge, a sour and slightly tart taste, a slightly sweet aftertaste, and a characteristic almond flavor; the moderate type is thought to have the best fragrance and taste [110,114,115,117]. This fermented product has long been used for its therapeutic properties. Since 1858, it has routinely been used in hospitals; approximately 23 hospitals in the former USSR reportedly use *koumiss* therapy, combined with antibiotic and other treatments. This fermented milk is believed to be useful in assisting the treatment of various neurological and digestive systems disorders; of tuberculosis, cardiovascular, respiratory and urinary diseases; and also of cancer, AIDS, herpes, attention deficit hyperactivity disorder (ADHD), and insomnia. It is also considered an immune stimulator [109,115,118,119]. It is also used as raw matter for producing a high alcohol drink called Arkhi—it is manufactured in Mongolia by distillation and contains 10–15% alcohol [119]. Many researchers have used horse milk for making other types of fermented liquid products. Bornaz et al. [120] studied the fermentation kinetics during production of a fermented milk by using a mixed culture of *Lactococcus lactis* subsp. *lactis* and subsp. *cremoris*. A longer latency phase (284 min) and lower acidification rate (μmax = 0.0052 dpH min^−1^) was found in comparison with bovine milk (194 min and 0.0098 dpH min^−1^, respectively) due to the high presence of lysozyme; maximum demineralization of casein micelles started around pH 6.09 and 5.31, for horse and bovine milk, respectively. Di Cagno et al. [121] manufactured a series of fermented milk prototypes by using the classical yogurt starter mixture (*Lactobacillus delbrueckii* subsp. *bulgaricus* and *Streptococcus thermophilus*) in the presence of Na-caseinate, pectin, sucrose, and threonine at different concentrations and combinations among them. The interference of lysozyme on cell growth was reduced by heating milk at 90 °C for 3 min. Fortification with Na-caseinate, pectin, and threonine enhanced the rheological and sensory properties, and most of the samples maintained suitable microbiological, rheological, and sensorial features after 45 days of storage at 4 °C.

Also, donkey milk is traditionally used to produce koumiss-like products with similar characteristics to the horse product [119]. Recently, this milk has been proposed for the production of new functional milk drinks through fermentation using selected microorganisms with probiotic properties. The use of probiotic bacteria strains such as *Lactobacillus rhamnosus* AT 194 and CLT 2/2, and *Lactobacillus casei* LC 88 has revealed donkey milk’s capacity for fermentation and the high viability of the probiotic strains after fermentation and storage, until 30 days [122,123,124,125]. In addition to the common cultures for yogurt preparation, Perna et al. [126] have also used *Lactobacillus acidophilus* and *Lactobacillus casei*, obtaining a product with a lower lactose content, higher antioxidant activity, and good sensory quality in terms of appearance, flavor, texture, and overall quality. Aspri et al. [127] proposed the use of selected LAB isolated from raw donkey milk to produce a new functional dairy drink with anti-hypertensive, antimicrobial, and/or antioxidant activities. Recently, the production of koumiss at the industrial level has raised the problem of controlling the type of milk used. In fact, horse milk is more expensive than cow milk, and the possibility exists for producers and dealers to adulterate equine products with bovine items. A triplex real-time PCR was recently developed for the identification of bovine and equine DNA by employing developed primers and probes. The limits of detection of the method were 0.001 ng for milk and yogurt and 0.005 ng for sour soup and koumiss, respectively [128].

### 3.2. Equine Cheese

**Figure 1:**
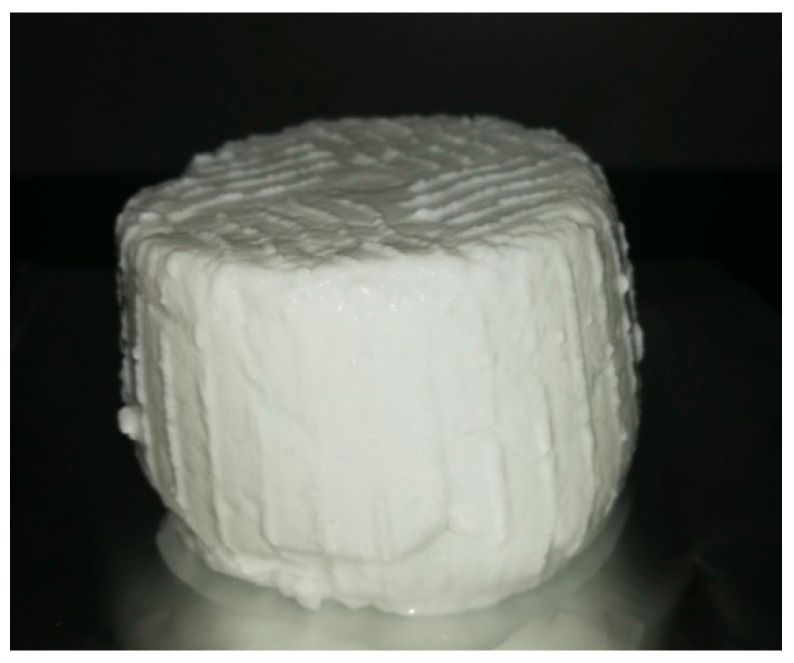
Fresh cheese from donkey milk obtained by coagulation with microbial rennet from *Mucor miehi*.

Making cheese from equine milk is considered not feasible due to the concerns in rennet coagulation discussed above. Nevertheless, several recent studies have shown it is possible to produce cheese from donkey milk by following dedicated technological approaches such as use of particular types of rennet, strong coagulating conditions, fortification with milk from other species, and addition of transglutaminase in order to better crosslink the milk proteins. The technological parameters applied in all these papers are summarized in Table 3. In 2015, Iannella [33] obtained a fresh cheese prototype by using camel chymosin. To make the cheese, milk was heated to 43 °C and inoculated with thermophilic starter cultures (Lactobacillus delbrueckii ssp. bulgaricus and Streptococcus thermophilus); after 1 h 30 min, pure camel chymosin (0.4 g 5 L^-1^ milk) was added. After 5 h incubation at 37 °C, the whey was drawn, and the curd was molded, obtaining 3.32% cheese yield. Unfortunately, the chemical characterization of the product was limited to determination of pH and total solids, whereas the sensory characteristics were not investigated. Sampajo [36] used cyprosin extract from Cynara cardunculus for coagulation at 32–35 °C, in the presence of CaCl_2_ (0.1% *w*/*v*). The results indicated a higher clotting activity for the plant aspartic protease in comparison to microbial rennet, and the cheese yield was 6.25%. A cheesemaking protocol was developed using calf rennet under strong technological conditions that allowed the obtaining of sufficiently firm curd for molding; the protocol yielded 5.9 kg cheese/100 L^−1^ milk. In this study, the coagulation parameters and in-vat operations were 0.3 g L^−1^ calcium chloride, milk acidification to pH 6.50, rennet (75% chymosin, 25% pepsin) at a dose of 1 mL L^−1^, coagulation temperature of 40 °C, and scalding to 46 °C after curd formation. On sensory evaluation, the cheese (a fresh type having 32.4% dry matter) proved to be friable and with a slightly soluble texture, moderately “gamy” with a “cooked milk” aroma, and markedly sweet and medium salty taste [34]. The same research group repeated the experimentation by employing microbial rennet from Mucor miehi and obtained a cheese (Figure 1) with the same characteristics but with higher yield (6.9%), probably due to the more favorable gross composition of the milk [35]. An extra hard cheese (six months ripening) was produced by fortifying donkey milk with goat milk (60:40 *v*/*v*), and adding calcium chloride (0.2 g L^−1^), commercial starter, and natural rennet (chymosin/pepsin, +96/4; 32 °C). The textural and sensory analysis revealed that the cheese was moderately hard and crumbly with a pronounced creamy, fatty, and acidic taste [129].

Faccia et al. [34] fortified donkey milk to produce fresh cheese, adding goat milk in ratios of 85/15 and 70/30 (*v*/*v*; donkey/goat milk), as did D’Alessandro et al. [130], also adding cow milk in a ratio of 70/30 (*v*/*v*; donkey/goat or cow milk). As expected, the addition of goat milk, and even more so cow milk, improved the coagulation parameters and cheese yield (donkey–goat milk mixture: 10.2–10.7%; donkey–cow milk mixture: 11.4%). In another study, the addition of microbial transglutaminase together with rennet was proposed as a fortification agent for donkey milk cheese making, evaluating different patterns of the enzyme addition. MTGase is known to lead to the formation of a network among proteins and high molecular weight peptides, modifying the products’ protein functional properties and rheological characteristics. The results showed that the enzyme, when added simultaneously with the rennet to acidified milk (pH 6.3; 42 °C), improved the firmness of the cur, and favored the syneresis process and the retention of milk proteins [131]. From the data in Table 3, it can be concluded that different types of rennet can be used for donkey milk cheesemaking, but the curd obtained is always weak and is only suitable for making fresh cheese. The use of calcium chloride helps coagulation, as does the addition of transglutaminase, which significantly shortens the total processing time. The main problem remains the high cost of the product, which could be overcome only by increasing the milk productivity of the animals.

### 3.3. Camel Fresh and Fermented Milk

Camel milk is mostly consumed in its fresh form or spontaneously fermented milk. Most of the reports made on milk focused on characterizing individual milk components, mainly proteins for their functionalities for processing or health benefits [132,133]. As a result of the increased demand, processed camel milk products are becoming popular [65,134] and are highly considered for health-related compositional properties, with particular reference to biologically active proteins [66,133]. However, due to difficulties in manufacturing cheese and yoghurts similar to those made from bovine milk, the camel milk products developed thus far are still limited [135,136]. There are many fermented camel milk products produced in different parts of the world, i.e., *gariss* in Sudan; *suusac* in East Africa, Kenya, and Somalia; *shubat* in Turkey, Kazakhstan, and Turkmenistan; and *dhaanan*/*ittitu* in Ethiopia [136,137,138,139,140]. The manufacturing procedure of these products is semi continue, using backslopping techniques with starter culture from spontaneous fermentation. However, there is a trend to standardize the starter cultures types for acidification in making *garis* and *suusac* [138,141].

In addition to the spontaneous microflora, fermentation of *gariss* is reported to be initiated by athe ddition of black cumin seed and onion bulbs [141]. Lactic acid bacteria (LAB) isolated from spontaneously fermented camel milk were characterized. Lactococci, lactobacilli, and pediococci were frequently identified in Ethiopian samples; while *Lactococcus, Lactobacillus, Enterococcus, Leuconostoc*, and *Streptococcus* species were reported for *garis* from Sudan [142,143]. *Suusac* from Kenya contained *Lactobacillus* and *Leuconostoc* species, as well as the yeast species *Candida, Geotrichum*, and *Rhodotorula* [144]. This variability in microflora might be due to uncontrolled fermentation in the production process and environmental conditions that promotes the growth of LAB species that become specific of the products. Spontaneously fermented camel milk in Ethiopia had poor safety standard [145]. There is a need to develop and commercialize specific starter cultures for specific products with different flavors and textures.

### 3.4. Camel Cheese

**Figure 2:**
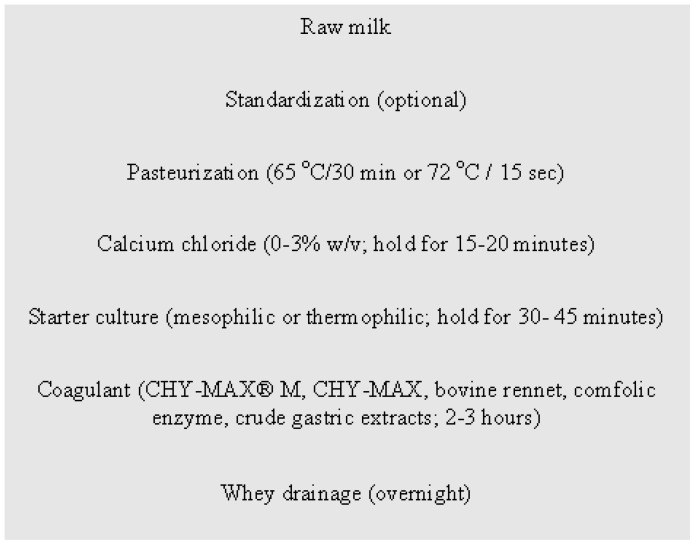
General scheme for manufacturing cheese from camel milk.

Production of cheese from camel milk has been reported to be tedious due to the unique qualitative and quantitative properties of proteins that cause long coagulation times and soft curd [67,70,139,146,147]. Making cheese from camel milk is an ongoing battle aimed to obtain a better quality, and continuous efforts are being undertaken for optimizing the technological parameters and taste. A study on the effect of the stage of lactation on cheese-making feasibility revealed that milk before 20th days of postpartum is not suitable for cheese making [148]. Milk concentrated two- and four-fold formed firmer gels than unconcentrated milk (2.45% protein) within a reasonable coagulation time (50 min) and enzyme dosage (35 IMCU/L); calcium addition at pH 6.00, had no significant effect [149]. Use of gastric extracts from abomasum of camels at different ages (one, three, and nine years) for making cheese was tested: the extract from older camels gave the best results, and the optimum flocculation time was obtained at pH 5.8 and 42 °C [133]. The effect of heating on cheesemaking is controversial: Kamal et al. [150] reported that preheating milk at 50 °C negatively affected the gelation properties, while preheating at 70 °C prevented the formation of rennet-induced milk gels. According to other studies, both in-vat (65 °C for 15 min) and heat-exchanger (71 °C for 15 s) pasteurization were compatible with cheesemaking [70,151]. Discrepant results could derive from different experimental conditions of heating or from differences in milk composition, including genetic variants; for cow milk, the effect of these parameters on coagulation is well known [152,153,154]. A general technological scheme for making cheese from camel milk is summarized in Figure 2. Most of the investigations conducted regarded soft cheese obtained by enzymatic coagulation. The acidity and composition varied with the manufacturing procedures, and the mean reported composition is 5.76 pH, 38.15 g/100 g total solids, 17.37 g/100 g protein, 16.41 g/100 g fat, and 2.23 g/100 g ash [155,156,157,158,159]. Overall, the mean values reported widely varied due to differences in coagulation time, curd strength, level of CaCl_2_, type starter culture used, and recipe followed during processing. The composition of camel Halloumi and Feta type cheese, and comparison of the recipes for their preparation were reported [159]. Fortification with sheep milk and coagulation with Camifloc rennet was a strategy to improve the processing properties of camel milk. Both the fortified and pure camel products were stored for 21 days at 37–40 °C in the whey, with acceptable sensory results. However, the change in microbiological load was not investigated [160]. Storage of soft cheese at 4 ± 1 °C and 18 ± 1 °C was monitored, resulting in increase in protein, fat, and total solid, but the product stored at 18 ± 1 °C could only be kept for maximum of six days [161]. Commercial chymosin (Chy-MaxTM with an activity of 600 IMCU mL^−1^) was tested at different levels in cheesemaking in order to evaluate cheese yield and quality. The most suitable level was 1.7 mL L^−1^, resulting in a cheese yield of 16.74 g 100 mL^−1^ [162]. Soft cheese (Domiati type) was made with variable percentage of salt, fat and type of starters (*Streptococcus thermophilus*, *Lactobacillus delbrueckii* ssp. *bulgaricus* and *Lactococcus lactis* ssp. *cremoris, lactis*, and *diacetylactis*). The cheese made with 3% added salt had higher yield (12.29 ± 1.63%), which was lower than the yield from cow and buffalo milk [151].

Ultrafiltration was applied to concentrate the milk and improve the cheesemaking properties and cheese quality. It resulted in the reduction of the doses of CaCl_2_, starter culture, and coagulant enzyme. The results showed a similar trend than the counterpart made from cow milk. Ultrafiltered cheese had high recovery of fat and protein, with acceptable sensory score than conventionally made cheese [163]. The manufacturing technology and compositional changes of semi-ripened brine-salted Halloumi-type and dry-salted Feta-type cheese and brine-ripened cheese were described. The evolution of chemical composition and of casein proteolysis during ripening follows a similar trend than the counterpart made from cow milk [159,164]. A few reports are available about the sensory characteristics of camel cheeses. Consumer acceptability of soft and semi-ripened cheese from camel milk were examined and resulted acceptable in five basic sensory attributes (appearance, color, taste, flavor, and texture) [156,157,162,165]; however, Benkerroum et al. [162] reported that the acceptability of soft cheese depended on concentration of coagulant and 1.7 mL L^1^ dose (1020 IMCU L^−1^) was chosen as optimal. Descriptive sensory analysis was applied to soft-brined Feta-type camel cheese: the sensory attributes were not specific and resembled most brined cheese made from milk of other species; the most important descriptors were salty, sour, and firm. Also, the volatile aroma compounds (VOCs) were not very different from those reported in the literature in similar bovine cheeses; similarly, the VOC profile was affected by ripening time and coagulant and NaCl levels [164].

### 3.5. Yak and Other Species Products

Reports on milk products from other species are very scarce and are unique to special regions of the world. Consequently, yak, reindeer, llama, alpaca, and moose can be considered as “marginal” dairy species. The most investigated is the yak, whose females (called *Nak* in Nepal language) are milked for 180 days beginning 3–4 weeks after calving. They are milked once a day in the morning, yielding approximately 1.36 (±0.34) liter of milk per day. Some of the milk is drunk fresh, but most of it is processed into yogurt, butter/ghee, and cheese [166].

The name Kurut refers to a group of yoghurts produced by natural fermentation of milk in China [167,168]. It is almost known in all regions of Qinghai as an indigenous fermented milk product with economic and nutritional importance, and both alcohol and lactic acid sensory attributes are its unique features [169]. Kurut from Qinghai is made in specially treated big jar for 7–8 days at 10–15 °C, and these conditions are necessary to produce enough acid, alcohol and flavour. The product contained greater numbers of LAB and yeast than other traditional fermented milks (LAB counts of 9.18 ± 0.851 log cfu/mL; yeast counts of 8.33 ± 0.624 log cfu/mL) [169]. *Lactobacillus delbrueckii* subspp. *bulgaricus* and *Streptococcus thermophilus* were the dominant microorganisms identified in Kurut samples [167], but a genomic study also revealed the relevant presence of *Lactococcus lactis* subspp *lactis*, *Lactobacillus helveticus*, and *Acetobacter* [170]. The chemical composition had 14.3, 5.37, 5.44, 2.34, and 0.86 g/100 g of total solids, fat, protein, lactose, and ash, respectively [169]. The VOC profile of yak fermented milk has been recently investigated by several authors. Fang et al. [171] proposed it as a tool for differentiating products made in five ecoregions in the Qinghai-Tibetan plateau. Although the VOC concentrations varied among the different ecoregions, the samples collected from geographically close ecoregions had similar composition. This finding might be related to the types of grassland and the microbiological characteristics. Specific connection between VOC and microbial species was reported for *Lactobacillus* (significant correlation with benzaldehyde, 2,3-pentanedione, ethanol, and ethylacetate), whereas *Streptococcus* and *Lactococcus* were associated with relatively low contents of volatile compounds [172].

*Chhurpi* or *churpi* is the name used for different types of cottage cheese manufactured in India, China and Nepal from different milks (cow, buffalo, or yak). The hard type is only made from yak or *dzomo* (a crossbreed of cow and yak) milk and is very popular among Tibetans, Nepali, and Mongolians. It can be ripened for a few years since is very stable; it has a very firm texture (about 68–90% total solids) containing calcium phosphate crystals and high level of protein (about 60–79%) [173,174]. It is manufactured from skimmed milk by precipitation of casein as a consequence of lactic fermentation (buttermilk is added as starter) or addition of alum salt (*Fitkiri*). Fermentation causes sour taste, the other method gives rise to a sweetish taste. In detail, after curdling the curd is wrapped tightly with a cloth and cured at room temperature (15–20 °C) for 2–3 days under pressure of heavy stones. Then, the cheese is sliced and dried in shade or over a wood fire oven and stored for long time. According to age, the cheese has different names: *chhursingba* (the younger), *chhur chirpen*, and *chhurpupu* (the older) [175,176]. Its production has not yet been standardized and the microbiological profile can widely change; the presence of potentially probiotic lactic acid bacteria has been reported [177]. *Qula* is another yak cheese manufactured in China; it has been a traditional food of Tibetan people for more than 1000 years and is usually produced in summer season by adding yogurt cultures to milk for spontaneous formation [178]. It presents a grainy and hard texture, with yellow or white colour. *Lactobacillus* and *Acetobacter* were the dominant bacteria genera in cheese samples taken from different part of China [179]. Probiotic activities of Lactobacilli strains isolated from *Qula* were screened by Zhang et al. [180], who found seven strains of *Lactobacillus* (three *actobacillus casei*, three *L. plantarum* and one *L. buchneri*) as good probiotic candidates, on the basis of tolerance against bile acid, simulated gastric and intestinal juices, antimicrobial activity, antibiotic resistance, and cell surface hydrophobicity. The fatty acid composition of cheese from yaks reared in the highlands of the Nepalese Himalayas (YC) was compared with that of cow Cheddar cheese (DC) purchased on the market. The results showed that YC had lower content of total medium-chain saturated fatty acids (C10:0–C16:0, 36.7% vs. 47.3%) and higher content of long-chain saturated fatty acids (C17:0–C26:0). The concentration of n-3 PUFA was 3.2 times higher than DC, and the ratio of n-3 to n-6 PUFA was 0.87 versus 0.20. The percentage of vaccenic acid in YC was 4.6 times higher than in DC, and the total conjugated linoleic acid (CLA) content was 2.3% of total fatty acids compared to 0.57% in DC [181].

Another interesting yak dairy product is Tibetan ghee, basically a clarified butter produced by a series of operations such as fermentation, heat clarification, and desiccation. Due to the attractive appearance, grainy and semisolid texture, pleasant odor, excellent taste, and the high proportion of polyunsaturated fatty acids, it has many culinary applications, such as dressing, cooking, and frying. A survey conducted on 50 samples taken from different areas revealed that the average fat content ranged from 71.68% to 93.3%, and only a small quantity of protein was present (0.51–1.81%). The acid and peroxide values varied from 0.02 mg/g to 1.30 mg/g and 0.07 meq/kg to 5.93 meq/kg, respectively. The content of minerals significantly varied with altitude level and region, as well as the fatty acid composition; the cause of the differences were hypothesized to be connected to the high unsaturated fatty acids level in the yak diets [182]. The influence of the diet was also found by Marquardt et al. [183], who also found differences connected to genotype: ghee made from hybrids (cattle-yak) milk had higher content of α-linolenic and linoleic acid, while yak ghee contained more saturated fatty acids and eicosapentaenoic, docosapentaenoic, and docosahexaenoic acids.

Reports on composition and manufacturing procedure of milk products such as cheese from reindeer, alpaca, moose, and others are very rare, in connection with the not fully domesticated status of all these animals, in particular for milk production. The available report for reindeer indicated possible curd formation and the easy production of cheese [184]. An historical study of the Sami people in Sweden reported that reindeer cheese was a source of income during the summer, but the manufacturing practices were not described [185]. Several journalistic reports are available on the web that mention small producers of reindeer cheese, but no sound scientific information can be found. The possibility of making cheese from alpaca milk was investigated—a semi-hard product was manufactured, whose chemical composition was characterized by 46.5% moisture, 41.8% protein, 24.4% fat on dry matter, and 18.9% ash. Due to poor fat content, the cheese had a hard texture, but the flavor was judged as pleasant; the cheese yield was 21.6 ± 1.9% [89]. Finally, the production of moose and llama cheeses were reported on the web. About 300 kg of cheese were obtained from three moose milked in ELK house in Sweden, but no details were given about the manufacturing procedure [186]. The same is for llama milk, which is reported to be occasionally transformed into a long-lasting cheese that is salty, rich, and heavy in texture. It seems that it can be found in small, local markets in the Andes region, where it is used as a dressing for local foods, like empanadas [187].

## 4. Conclusions

Minor milks and related products have long been relegated to a “dark” area of research due to poor economic significance. The increasing interest toward ethnic foods and alternative milk products with functional properties is now shining a light on them. Several traditional products have been scientifically characterized, and it appears that they could be well valorized in niche markets, together with new types that are increasingly being developed by researchers. However, much work is still needed to create specific production chains; the main challenges are to standardize the technological protocols and to complete the chemical-nutritional and microbiological information. These challenges should be faced with an interdisciplinary approach, in which animal scientists and food technologists interact for increasing the production volume in connection with some particular qualitative aspects.

## Figures and Tables

**Table 1:** Compositional aspects of technological relevance of minor milks (average values from the cited literature); n.c. = not considered.

	Horse	Donkey	Camel	Yak	Cow	Human
Total solids %	10.2	8.8	12.5	16.0	12.7	12.4
Lactose %	6.4	6.9	4.5	5.3	4.8	7.0
Protein %	2.1	1.7	3.3	4.2	3.4	0.9
Casein/whey proteins	1.1	1.3	1.7	4.5	4.7	0.4
Casein micelle (ø, nm)	255	298	380	220	182	64
αs1 (% total casein)	46	25	22	31	38	12
αs2	1	2	10	10	10	-
β	46	70	65	48	36	65
κ	<1	<1	3	11	13	23
others	6	3	n.c.	n.c.	3	n.c.
Fat %	1.2	0.4	3.8	5.6	3.7	3.8
Fat globule *(*ø, µm)	2.5	1.9	3.0	4.4	3.9	4
Total calcium (mmol L^−1^)	15	20	25	33	27	7.5
Colloidal calcium (% of total)	60	45	70	58	67	36

**Table 2:** Somatic cell counts (SCC, ×1000 mL^−1^) and total bacterial counts (TBC, ×1000 mL^−1^) of milk from minor dairy species. Rf = reference.

Milk	n	SCC (Mean)	SCC (Min–Max)	TBC (Mean)	TBC (Min–Max)	Rf
Horse	260	n.r.	n.r.	14.1	n.r.	[52]
Horse	300	62	41–194	n.r.	33–51	[54]
Donkey	10	<50	1.4–600	n.r.	0.01–0.25	[47]
Donkey	88	n.r.	n.r.	15.2	0.01–90	[50]
Donkey	6	n.r.	n.r.	21.9	9.3–51.3	[51]
Donkey	152	n.r	n.r.	n.r.	0.63–10	[48]
Donkey	1 BM	n.r.	n.r.	69.2	n.r.	[55]
Camel	458	404	251–562	5.2	4.0–6.0	[57]
Camel	33	n.r.	97–720	n.r.	n.r.	[58] *
Camel	38	n.r.	25–331	n.r	n.r.	[59]
Camel	10	69	28–121	n.r.	n.r.	[60]
Camel	84	n.r.	n.r.	7.1	0–25	[61]
Camel	1 BM	n.r.	n.r.	31.6	6–150	[62]
Camel	34 BM	n.r.	n.r.	100	1–14,125	[63]
Yak	24	n.r.	n.r.	380	18.2–2570	[64]

n = number of samples; Rf = reference; n.r. = not reported; BM = bulk milk; * = results categorized on the basis of California Mastitis Test reaction.

**Table 3:** Technological conditions for manufacturing cheese from donkey milk.

Milk	Rennet	T (°C)	CaCl_2_%	Milk pH	CT	Curd Firmness	Cheese TS%	Y%	Rf
D	C	32	0.10	7.4	45′	weak	n.r.	6.25	[36]
D	MR	32–35	0.10	7.4	n.r.	very weak	n.r.	n.r.	[36]
D	MR	42	0.03	6.3	42′	weak	34.2	6.9	[35]
D	CR	40	0.03	6.5	3 h	weak	32.4	5.9	[34]
D	CmR	37	-	7.06	5 h	clotted precipitate	35,6	3.32	[33]
D	MR + TG	42	0.03	6.3	34′	weak	36.3	6.91	[130]
D/G (60/40)	CR	32	0.02	7.2	45′	firm	61.5 *	4.0 *	[129]
D/G (85/15)	CR	40	0.03	6.5	2 h	soft	36.7	8.1	[34]
D/G (70/30)	CR	40	0.03	6.5	1 h 40′	firm	38.2	10.2	[34]
D/C (70/30)	MR	40	0.03	6.3	n.r.	firm	n.r.	11.4	[131]

D = donkey; G = goat; C = cow; CT = cheesemaking time (from rennet addition to cheese moulding); TS = total solids; Y = yield; Rf = reference; C = cyprosin from *Cynara cardunculus*; MR = microbial rennet from *Mucor miehei*; CR = calf rennet; CmR = camel rennet; TG *= transglutaminase*; * = extra-hard cheese after 6 months ripening; n.r. = not reported.
